# *Bifidobacterium bifidum* SAM-VI Riboswitch Conformation Change Requires Peripheral Helix Formation

**DOI:** 10.3390/biom14070742

**Published:** 2024-06-23

**Authors:** Wenwen Xiao, Guangfeng Liu, Ting Chen, Yunlong Zhang, Changrui Lu

**Affiliations:** 1 College of Biological and Medical Engineering, Donghua University, Shanghai 201620, China; wenwen_xiao@yeah.net (W.X.); chenting@dhu.edu.cn (T.C.); zhyl@dhu.edu.cn (Y.Z.); 2 National Center for Protein Science Shanghai, Shanghai Advanced Research Institute, Chinese Academy of Sciences, Shanghai 201204, China; liuguangfeng@sari.ac.cn

**Keywords:** riboswitch, S-adenosyl-methionine, conformational dynamics, SAM-VI riboswitch, 3D modeling, SAXS, SHAPE

## Abstract

The *Bifidobacterium bifidum* SAM-VI riboswitch undergoes dynamic conformational changes that modulate downstream gene expression. Traditional structural methods such as crystallography capture the bound conformation at high resolution, and additional efforts would reveal details from the dynamic transition. Here, we revealed a transcription-dependent conformation model for *Bifidobacterium bifidum* SAM-VI riboswitch. In this study, we combine small-angle X-ray scattering, chemical probing, and isothermal titration calorimetry to unveil the ligand-binding properties and conformational changes of the *Bifidobacterium bifidum* SAM-VI riboswitch and its variants. Our results suggest that the SAM-VI riboswitch contains a pre-organized ligand-binding pocket and stabilizes into the bound conformation upon binding to SAM. Whether the P1 stem formed and variations in length critically influence the conformational dynamics of the SAM-VI riboswitch. Our study provides the basis for artificially engineering the riboswitch by manipulating its peripheral sequences without modifying the SAM-binding core.

## 1. Introduction

Riboswitches typically cis-regulate downstream genes and situate in the 5′-UTR region (untranslated regions) upstream of mRNAs’ coding region. They function independently of protein factors, relying solely on responding to various environmental changes such as coenzymes [1,2,3,4,5,6,7,8,9,10,11,12,13,14], FFnucleotide derivatives [15,16], signaling molecules [17,18,19,20,21], ions [22,23,24], amino acids [25,26,27,28], and other metabolites. Since its first publication in 2002, over 55 riboswitches have been reported and validated [29]. Riboswitches control several critical metabolic processes in pathogenic bacteria, including but not limited to the thiamine pyrophosphate (TPP) riboswitch in *Bacillus anthracis* [30], the glmS riboswitch in *Staphylococcus aureus* [31], the Mn-sensing riboswitch [24], the glycine riboswitch in *Streptococcus* spp. [32,33], and the glycine riboswitch in *Clostridium difficile* [34]. Riboswitches typically consist of two main components: the aptamer domain and the expression platform [35,36]. Aptamers possess highly conserved sequences, enabling them to form complex three-dimensional (3D) structures that specifically bind to particular cellular metabolites, which in this context function as ligands [37,38]. Upon ligand binding, the aptamer and the downstream expression platform of the riboswitch undergo conformational changes [39]. This process affects the expression of genes encoded downstream of the riboswitch at the transcriptional decay or translation initiation of many critical metabolic pathways [40].

S-adenosyl-methionine (SAM) participates in numerous metabolic processes [41], primarily serving as a ubiquitous methyl donor in biochemical methylation reactions across all organisms [42]. The SAM riboswitch is one of the most abundant riboswitches that bind to SAM, with high affinity and selectivity. Given the significance of SAM metabolites, SAM riboswitches control many critical biochemical pathways across diverse bacterial species. Seven distinct families of SAM riboswitches to date [6,7,8,9,10,11,12,13,43] have had atomic-resolution three-dimensional structural models of their aptamer domains resolved [43,44,45,46,47,48,49,50]. Each SAM riboswitch family has a well-organized tertiary core scaffold to support its unique ligand-specific binding pocket. Studies on the distribution, structure, ligand recognition, and gene regulatory mechanisms of these SAM riboswitch families will advance the understanding of their evolutionary prospects and potential applications.

**Figure 1:**
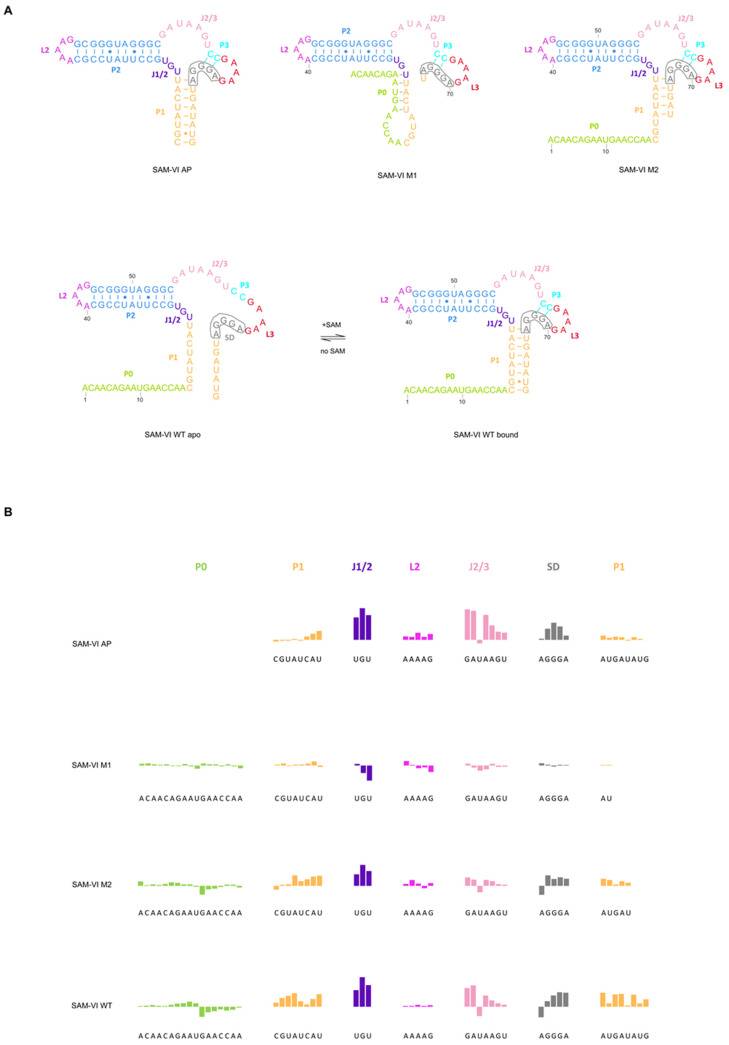
Secondary structure and SHAPE probing of the SAM-VI riboswitch. (**A**) Secondary structure of the wild-type SAM-VI riboswitch (apo and bound), the SAM-VI AP RNA (the aptamer domain of the SAM-VI riboswitch, C18-G81), the SAM-VI M1 RNA (deleting six nucleotides from the 3′ end of the SAM-VI riboswitch, which does not contain the WT P1), and the SAM-VI M2 RNA (deleting three nucleotides from the 3′ end of the SAM-VI riboswitch). The Shine-Dalgarno sequence is highlighted (gray wireframe). (**B**) SHAPE was carried out for the SAM-VI AP RNA (row 1), the SAM-VI M1 RNA (row 2), the SAM-VI M2 RNA (row 3), and the SAM-VI WT RNA (row 4). The quantified reactivity profile revealed structural changes upon SAM binding extracted from the raw data shown in each row. The coloring of the SAM-VI riboswitch with the SHAPE signal is consistent with the secondary structure in (**A**). Upward bars represent protected residues due to SAM-induced structure formation. Downward bars represent exposed bases upon SAM-binding. Residue numbers and corresponding structural motifs are indicated.

Even with the resolved crystal structures, riboswitches exhibit highly flexible structural dynamics. Upon ligand binding, riboswitches undergo dynamic conformational changes that modulate downstream gene expression by promoting or inhibiting transcription or translation. The dynamics of riboswitches involve a delicate interplay between structural rearrangements, ligand recognition, and interactions with cellular machinery. Understanding the dynamics of riboswitches is crucial to elucidating their regulatory roles in various cellular processes and developing potential therapeutic interventions targeting these unique RNA elements. However, these conformational changes proved challenging to capture. Related research has provided ample foundations for further work [40,51,52,53,54,55,56,57]. The SAM-VI riboswitch is a newly discovered class of SAM riboswitches. The Shine-Dalgarno (SD) sequence within the SAM-VI riboswitch shares sequences with P1 and P3. In the absence of SAM, P1, and P3 exhibit greater flexibility, allowing the SD sequence to be exposed and initiate translation. Conversely, in the presence of SAM, the SAM-VI riboswitch specifically binds to SAM, stabilizing P1 and P3, which sequesters the SD sequence, preventing its interaction with the ribosome and consequently inhibiting translation (Figure 1A). Consequently, we propose to use the SAM-VI riboswitch [44] as a model system to study its molecular dynamics.

In this study, we have analyzed the *B. bifidum* SAM-VI riboswitch and its variants using 3D structure prediction, SHAPE probing, SAXS, and ITC to elucidate their unique conformations and dynamics in the presence and absence of SAM. *B. bifidum* strains have beneficial effects, including antibacterial features against pathogens like *Helicobacter pylori* [58,59,60]. They also affect the host human immune system [61,62,63,64]. We found that the length of P1 significantly affects the conformational dynamics of the SAM-VI riboswitch. Modulating the *B. bifidum* SAM-VI riboswitch activities could alleviate inflammatory activities associated with certain chronic gut dysfunctions [63,64].

## 2. Results

### 2.1. Design of the SAM-VI Riboswitch and Its Variant Constructs for Structural Studies

This study uses the wild-type SAM-VI RNA from the 5′-UTR of the *metK* gene of *Bifidobacterium bifidum* as model system. To comprehensively investigate the conformation of the SAM-VI riboswitch and understand its gene regulatory mechanism, we designed three variants: SAM-VI AP, with P0 (ACAACAGAAUGAACCAA) removed and containing only the aptamer domain; SAM-VI M1, with six nucleotides (GAUAUG) deleted from the 3′ end, this variant does not contain the WT P1; and SAM-VI M2, with three nucleotides (AUG) deleted from the 3′ end. The SAM-VI M1 and SAM-VI M2 variants serve as slightly extended intermediates in the transcriptional process of the SAM-VI riboswitch. The sequence and secondary structure of these constructs are shown in Figure 1A. These mutations would help us understand the effect of peripheral sequences on the riboswitch dynamics.

### 2.2. SHAPE Analysis of SAM-Induced Conformational Dynamics in the SAM-VI Riboswitch

We utilized SHAPE chemical probing methods to characterize the ligand-induced conformational dynamics within the SAM-VI riboswitch. We assessed the effect of ligand binding on the SAM-VI riboswitch by examining both SAM-bound and SAM-free states. Figure 1B depicts SHAPE probing results of the SAM-VI riboswitch, including SAM-VI AP (row 1), SAM-VI M1 (row 2), SAM-VI M2 (row 3), and the wild-type SAM-VI riboswitch (row 4).

Since the upward bars in SHAPE results represent gained stability upon ligand binding and vice versa, SAM-binding can stabilize the SAM-VI aptamer and the M2 and WT constructs, with the exception of the M1 mutation.

The SHAPE reactivity of the aptamer of SAM-VI WT (Figure 1B, row 4) is similar to that of the aptamer alone (Figure 1B, row 1). The SHAPE reactivity values of the P0 (green bars), L2 (purple bars), and P2 stem remain stable in both the ligand-bound and ligand-free states. Conversely, residues near the SAM-binding pocket, including J1/2 (purple-blue bars) and J2/3 (pink bars) exhibit higher SHAPE reactivity in the ligand-free state, suggesting that in the absence of a ligand, these sequences show higher conformational dynamics, and hence disrupt the SAM-binding pocket. Consequently, the SD sequence (AGGGA) exhibits high SHAPE reactivity during the ligand-free state (Figure 1B). Distinct from SAM-VI AP, the P1 (orange bars) shows significant SHAPE reactivity, meaning these sequences become stable in the presence of ligands, while P0 (green bars) shows no change. Overall, SAM binding does not affect the stability of P0, L2, and P2, but it does stabilize the sequences at P1, the three-stem junction, and SD.

We designed the M1 and M2 mutants to systematically disrupt P1 to investigate its role in riboswitch function and dynamics. The M1 mutant, having no P1, exhibits no response to SAM (Figure 1B, row 2). Specifically, residues within the ligand-binding pocket exhibit no change in SHAPE reactivity across the entire sequence. This result demonstrates that the SAM-VI M1 has no conformational change upon the addition of SAM.

The M2 mutation, with a shorter P1 helix than the wild-type, partially rescued the riboswitch function. The SHAPE reactivity of SAM-VI M2 exhibits a pattern similar to that of the SAM-VI WT; however, the SHAPE reactivity values of corresponding residues decrease (Figure 1B, row 3). Therefore, we hypothesize that ligand binding induces the conformational change in the SAM-VI M2 variant, albeit with a weaker affinity than the SAM-VI WT.

### 2.3. SAXS Reveals SAM-Induced Conformational Changes of the SAM-VI Riboswitch

**Figure 2:**
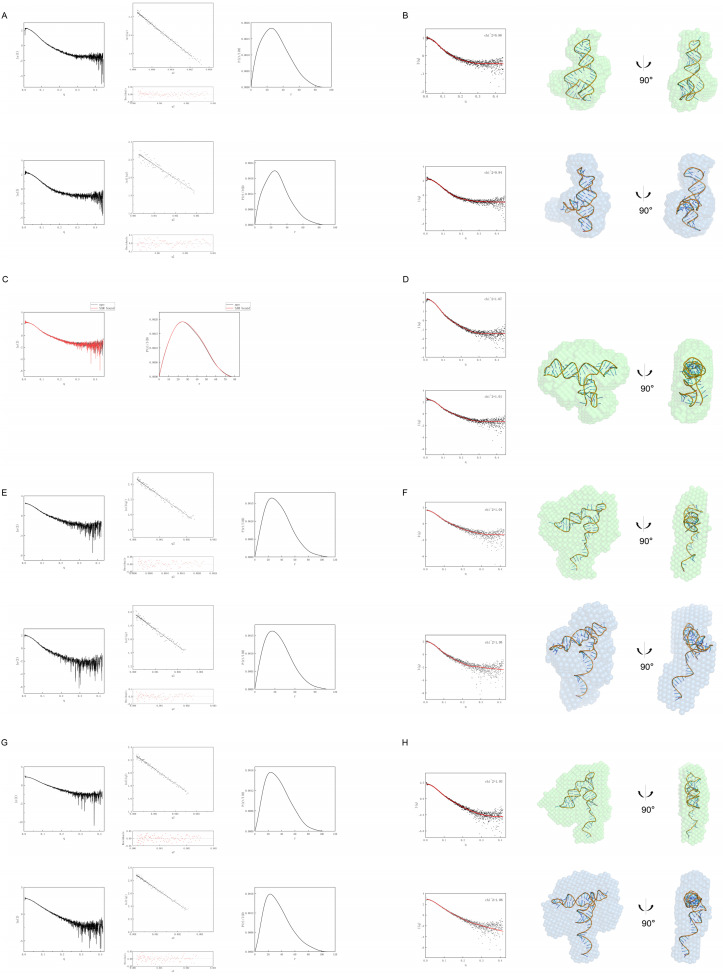
Conformational analysis by Small−Angle X−ray Scattering (SAXS) for the SAM−VI riboswitch and its variants in the presence and absence of ligand. (**A**,**B**) are SAXS data for the SAM−VI AP RNA. (**C**,**D**) are SAXS data for the SAM−VI M1 RNA. (**E**,**F**) are SAXS data for the SAM−VI M2 RNA. (**G**,**H**) are SAXS data for the SAM−VI WT RNA. (**A**,**E**,**G**) Comparison of experimental scattering profiles (left), Normalized P(r) analysis (right), and Guinier plot (middle) for ligand−free RNA (upper series) and ligand−bound RNA (lower series). (**C**) Comparison of experimental scattering profiles (left) and Normalized P(r) analysis (right), the ligand−free RNA (black) and the ligand−bound RNA (red). (**B**,**F**,**H**) The theoretical scattering curve of the predicted atomic structure (red) was compared to the experimental scattering curves (black) by CRYSOL. Low−resolution bead models calculated by DAMMIF from SAXS data. Predicted atomic models of the RNA docked inside the SAXS bead models. The ligand−free RNA (upper series) is green, and the ligand−bound (lower series) RNA is blue. (**D**) Comparison of theoretical scattering curves (red) with experimental scattering curves (black) of RNA−predicted atomic structures by CRYSOL (left). Docking of the RNA−predicted atomic models within the SAXS bead model calculated by DAMMIF (right).

To obtain the molecular envelopes of the SAM-VI riboswitch, we performed size-exclusion small-angle X-ray scattering (SEC-SAXS) on both the ligand-free and the ligand-bound states to detect ligand-dependent global RNA conformational changes. The SAXS curves and corresponding 3D models of the SAM-VI riboswitch and its variants in the presence and absence of SAM are shown in Figure 2.

Overall, SAM-binding reduces the Guinier radius of gyration (Rg) of the SAM-VI AP and M2 variants. Based on SAXS analysis, the Rg for the ligand-free state of the SAM-VI AP is 23.54 Å, larger than the value of 21.75 Å measured for the ligand-bound state. Meanwhile, the Guinier radius of gyration (Rg) for the ligand-free state of the SAM-VI WT is 26.50 Å, similar to the value of 26.79 Å measured for the ligand-bound state. By comparison, the Rg for the ligand-free state of the SAM-VI M2 is 27.62 Å, larger than the value of 26.58 Å measured for the ligand-bound state. Next, to verify these conformational changes, we fitted predicted 3D models (see Methods for details) of the ligand-free and -bound states of SAM-VI AP, SAM-VI M2, and SAM-VI WT to the SAXS-generated 3D envelopes. CRYSOL results show that the ligand-free and -bound state models agree with the experimental scattering data, and the results of the dockings are shown in Figure 2B,F,H. Comparing the three sets of 3D bead models in the ligand-free and ligand-bound states, we found that the ligand-bound bead model showed compression between J1/2 and J2/3. The angle between P1 and P3 becomes smaller for the ligand-bound state compared with the apo state in SAM-VI M2, and P1 in the ligand-bound state swings to be nearly perpendicular to P3 in SAM-VI AP and SAM-VI WT.

The combined SHAPE probing and SAXS results led us to conclude that in the absence of SAM, the P1 and SD sequences in SAM-VI M2 and SAM-VI WT show more conformational flexibility and solvent accessibility, allowing partial exposure of the SD sequence for ribosome binding and subsequent translation initiation. Upon the incorporation of SAM, the P1 and SD sequences become stabilized, rendering the SD sequence inaccessible to the ribosome and thus shutting down translation.

Meanwhile, the M1 mutation abolishes this conformation change. We predicted a 3D model of the SAM-VI M1 using RNAComposer. The CRYSOL results indicate that the two experimental curves separately match the same predicted 3D model (Figure 2D). The 3D model matched the SAXS-calculated bead model in both angle and size. We found that instead of forming the P1 stem, the SAM-VI M1 appeared to form a P0 stem at the 5′ end and a P3 stem at the 3′ end. The experimental scattering curves of SAM-VI M1 in the ligand-free and -bound state were indistinguishable, and the particle pair distribution function P(r) curves almost overlapped (Figure 2C). Therefore, we conclude that these conformation changes require P1 formation, without which SAM-binding does not occur and the resulting RNA construct does not respond to ligands.

Our fitted 3D structures showed that when a P1 stem forms, the conformation of the SAM-VI riboswitch contains a pre-organized ligand-binding pocket. SAM selectively stabilizes this conformation through ligand-mediated tertiary interactions and drives the conformational equilibrium toward the bound state.

### 2.4. Calorimetric Analysis of Ligand Binding by the SAM-VI Riboswitch

**Figure 3:**
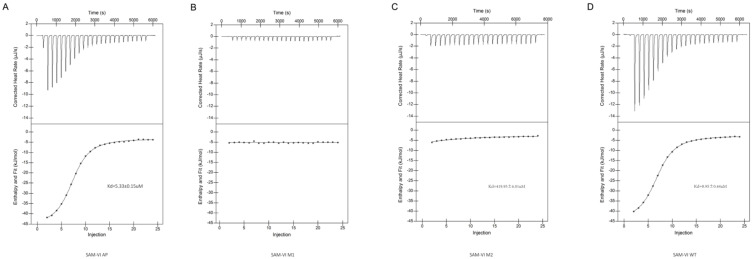
Isothermal titration calorimetric measurement of the SAM−VI riboswitch and variants. A solution of ligand was titrated into the SAM−VI AP RNA (**A**), the SAM−VI M1 RNA (**B**), the SAM−VI M2 RNA (**C**), and the SAM−VI WT RNA (**D**) separately; the heat evolved was measured. In each case, the upper panel shows the raw data for sequential injections. The lower panels present the integrated heat data fitted (where possible) to a single−site binding model.

Last but not least, we used isothermal titration calorimetry (ITC) to verify the affinity between the ligand and our SAM-VI riboswitch constructs (Figure 3). A ligand was titrated into different RNAs at 25 °C, and the heat changes were measured. Titration of SAM into the SAM-VI AP and SAM-VI WT resulted in the observation of exothermic binding with affinities of 5.33 and 8.95 μM, respectively. However, no significant calorimetric changes were observed when SAM was titrated into SAM-VI M1. Titration of SAM into SAM-VI M2 resulted in two orders of magnitude weaker calorimetric modifications than the SAM-VI WT, with a dissociation constant (Kd) of 419.95 μM. The results of isothermal titration calorimetry were matched with SHAPE probing and SEC-SAXS. Based on the ITC results, we also determined that the free energy change (ΔG) of SAM-VI M2 and SAM-VI WT binding to the ligand SAM was 19.28 kJ·mol^−1^ and 28.82 kJ·mol^−1^. The disparity in free energy changes explains the variation in affinity between the two RNAs for the ligand.

## 3. Discussion

In this study, we investigated the ligand-free and ligand-bound states of the SAM-VI riboswitch and its variant structures using a 3D structure prediction server, SAXS, and SHAPE probing. Our study revealed that in the ligand-bound state, the aptamer of the SAM-VI riboswitch resembles a twisted letter “T” and is stable in solution. Residues on J1/2 and J2/3 constitute the ligand binding pocket, consistent with previous studies [65]. Comparing the two sets of 3D bead models of SAM-VI WT and SAM-VI AP in the ligand-free and ligand-bound states, we find that the bead models in the ligand-bound state show compression at the ligand-bound pockets. Specifically, the previously flexible single-stranded J1/2 and J2/3 became stable upon ligand binding, and our data support previous results [65,66]. SAM binding caused compression of the conformation of the SAM-VI riboswitch at the three-way junction of the aptamer domain, which caused a pronounced oscillation of the P1 stem until it was perpendicular to the P3 stem and stabilized the structure of both the P1 and P3 stems. Our study revealed that the P0 stem of the SAM-VI riboswitch is different from that described in previous studies [65,66]. The structure of P0 is probably not conserved in different species and undergoes some flexible changes. Through comparison between the *B. angulatum* and *B. bifidum* SAM-VI riboswitch structures and functions, we concluded that despite some minor differences, they exhibit identical biochemical functions in vitro. We also concluded that a combination of conserved tertiary interactions within the riboswitch governs its structural integrity and conformational dynamics.

This study characterized two mutants of SAM-VI. The SAM-VI M1 does not contain a P1 stem and appeared to form a P0 stem at the 5′ end and a P3 stem at the 3′ end. This conformation cannot form a stable ligand-binding pocket, indicating that the SAM-VI M1 variant cannot bind to a ligand to regulate functions. The M2 mutation containing a short P1 partially rescued the riboswitch function and underwent conformational changes in response to SAM binding. Specifically, SAM binding caused compression of the conformation of the SAM-VI M2 at the three-way junction of the aptamer domain, which caused a pronounced oscillation of the P1 stem. The results of mutational analyses suggest that conformational changes in the SAM-VI riboswitch require peripheral helix formation. Earlier studies also found that variable sequences outside the ligand-binding core critically influence the conformational dynamics of the riboswitch. The results of mutational analyses suggest that conformational changes in the SAM-VI riboswitch require peripheral helix formation [40,51,67,68,69].

**Figure 4:**
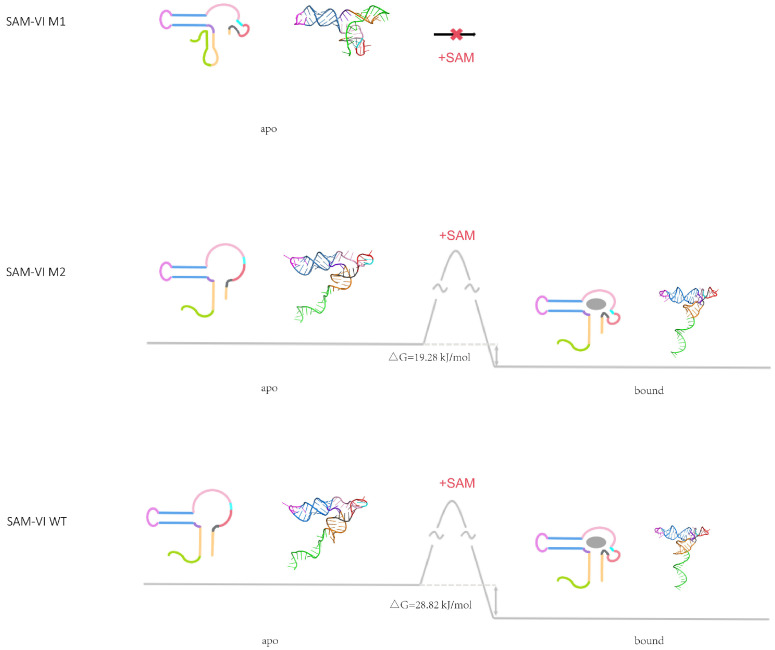
Proposed free energy landscape of the SAM-VI riboswitch binding to SAM. Proposed secondary structures and the 3D models of SAM-VI riboswitch are shown. Energetic differences calculated from SAM-binding Kd are marked. The color scheme is the same as that in Figure 1. Gray trajectory: the folding path of SAM-VI RNA with SAM. The SAM is shown as a gray sphere.

In summary, our combined chemical probing, SAXS, and ITC data defines a transcription-dependent conformation model for *B. bifidum* SAM-VI riboswitch (Figure 4). A P0 is formed at the 5′ end of the SAM-VI M1 variant, while the P1 stem is not produced. The overall structure of the SAM-VI M1 variant is flexible and unable to bind ligands. Consequently, its conformation remains unchanged in the presence or absence of SAM. As transcription proceeds, SAM-VI M2 and SAM-VI WT are produced sequentially and the P0 and P3 stems are unwound. In the absence of SAM, the P1 and P3 stems are relatively flexible, and the SD sequence is exposed for ribosome binding and subsequent translation initiation. Upon binding to SAM, the three-stem junctions are compressed and the P1 and P3 stem become stabilized, while the SD sequences are enclosed and inaccessible to the ribosome, thereby shutting down translation. SAM-VI M2 and SAM-VI WT bind to SAM but with different affinities. We observed a difference in the free energy change (ΔG) for SAM-VI M2 and SAM-VI WT binding to SAM of 19.28 kJ·mol^−1^ and 28.82 kJ·mol^−1^, respectively. The large disparity in free energy change offers an explanation for the variance in ligand affinity between the two RNAs. The above results indicate that both the presence and the absence of the P1 stem and length variations critically influence the conformational dynamics of the SAM-VI riboswitch.

Finally, our study provides a basis for artificially engineering the riboswitch by manipulating its peripheral sequences without modifying the SAM-binding core. Such engineering could prove useful in building artificial molecular sensors. In addition, since disrupting these peripheral regions practically disables the riboswitch, such an idea could also generate new ways to fight pathogens whose critical metabolic pathways rely on riboswitch regulation.

## 4. Materials and Methods

### 4.1. RNA Preparation

The SAM-VI riboswitch utilized in our research was derived from the *metK* gene of *Bifidobacterium bifidum*. In addition to the full-length and aptamer domain sequences of the wild-type SAM-VI riboswitch, we designed variants with different lengths named SAM-VI M1 and SAM-VI M2. For SAXS experiments, a T7 RNA polymerase binding site was incorporated at the 5′ end of the RNA sequence, and an HDV self-cleaving ribozyme was appended to the 3′ end. For SHAPE analysis, the T7 RNA polymerase binding site and 5′ linker sequence were sequentially appended to the 5′ end, while the 3′ linker and reverse transcriptase binding sites were added to the 3′ end. All constructs were cloned into PUC-SP vectors and synthesized by Sangon Biotech, Shanghai, China. The RNA was synthesized via in vitro transcription using T7 RNA polymerase and purified using a 12% Urea-PAGE gel. Subsequently, the target RNA was eluted into ddH_2_O, and concentration was achieved by centrifugation at low temperatures using Millipore centrifugation columns (Merck KGaA, Darmstadt, Germany). The RNA was annealed in a buffer containing 25 mM Tris-HCl (pH 7.5), 40 mM NaCl, and 5 mM MgCl_2_. It was heated at 65 °C for 10 min, then mixed with SAM to a final concentration of 1 mM (or no SAM added), incubated at 37 °C for an additional 5 min, and finally cooled on ice.

### 4.2. SHAPE Probing Analysis

A total of 9 μL of RNA solution (11 μM) was mixed with 1 μL of 1M7 (100 mM), and the mixture was then incubated for 10 min at 30 °C. The modified RNA was recovered by adding 446 μL of ethanol precipitation buffer, which contains 45 mM NaCl, 450 mM EDTA, 80% absolute ethanol, and 0.033 mg/mL glycol-blue. After precipitation at −80 °C for 30 min, the RNA was pelleted by centrifugation at 12,000 rpm for 30 min at 4 °C. The pellet was recovered and resuspended in 10 μL of ddH_2_O. The SHAPE experiment was conducted following procedures described elsewhere [70,71,72]. Reverse transcription was carried out using a FAM-labeled DNA primer. The cDNA fragments generated by reverse transcription were detected using capillary electrophoresis. Data analysis was performed using the ShapeFinder software (version 1.0) [73,74]. SHAPE reactivity was determined following normalization and scaling procedures [40,51].

### 4.3. Prediction of the Three-Dimensional Structure of the SAM-VI Riboswitch and Variants

Since there is no *Bifidobacterium bifidum* SAM-VI riboswitch crystal structure, we predicted its 3D structure in the absence of a ligand using RNAComposer (version 1.0), an automated RNA structure 3D modeling server [75]. Then combining the known SAM-bound crystal structure of *B. angulatum* and sequence constraints obtained by our SHAPE probing, 3D models of the ligand-bound *Bifidobacterium bifidum* SAM-VI riboswitch were predicted using RNAComposer. The 3D structures of the variants were obtained in the same way. The final models were modified with CRYSOL [76] (Version 3.2.1 (r14886)) [77] and the crystal structure of *B. angulatum*.

### 4.4. Small-Angle X-ray Scattering Experiments and Data Analysis

Small-angle X-ray scattering data were collected at the BL19U2 beamline at the National Facility for Protein Science Shanghai (NCPSS) and Shanghai Synchrotron Radiation Facility (SSRF). To obtain robust structural information from SAXS data, we leveraged the benefits of online size-exclusion chromatography (SEC) coupled with SAXS in our study. Size-exclusion chromatography (SEC) was conducted using a Superdex 75 Increase 10/300 GL column (Cytiva, Marlborough, MA, USA), which was equilibrated with a buffer containing 25 mM Tris-HCl (pH 7.5), 40 mM NaCl, 5 mM MgCl_2_, and either 1 mM SAM or no SAM. The concentration of the RNA samples ranged from 5 to 8 mg/mL. The wavelength was set to 1.033 Å. The SAXS data were analyzed using the BioXTAS RAW software (Version 2.4.1) [78]. All 2D images were converted into 1D SAXS curves. Buffer scattering was subtracted from sample scattering using PRIMUS [79] (Version 3.2.1 (r14886)). Pair distribution functions (P(r)) and the maximum sizes (D_max_) of the particles were calculated using the Guinier and GNOM methods [77,80]. We used DAMMIF/DAMMIN (Version 3.2.1 (r14886)) and DAMAVER (Version 3.2.1 (r14886)) to generate bead models [81,82,83], which were visualized using PYMOL (Version 3.8.5) [84]. The scattering profiles of the atomic models were assessed using CRYSOL (Version 3.2.1 (r14886)) and aligned with the experimental data [76]. Finally, SUPCOMB/CIFSUP (Version 3.2.1 (r14886)) aligned the bead models with predicted 3D structures [85].

### 4.5. Isothermal Titration Calorimetry

Titrations were conducted at 25 °C using a Nano ITC (TA Instruments, New Castle, DE, USA). Prior to titration, the RNA buffer was exchanged with an ITC buffer containing 25 mM Tris-HCl (pH 7.5), 40 mM NaCl, and 5 mM MgCl_2_ by centrifugation at 4 °C using Millipore centrifugal columns (Merck KGaA, Darmstadt, Germany). SAM was prepared in the same buffer at concentrations ranging from 0.6 to 2 mM. Solutions were degassed for 10 min prior to loading. The sample cell was filled with 950 μL of RNA solution. SAM was injected at a volume of 4 μL for the first injection and 10 μL for each of the subsequent 23 injections, with an injection interval of 200–250 s. Integrated heat data were analyzed using both the blank (constant) model and an independent model via NanoAnalyze Software (version 3.12.5), provided by the manufacturer. The first data point was excluded from the analysis. All ITC titration experiments were repeated independently (three replicates in total).

## Data Availability

The original contributions presented in the study are included in the article, further inquiries can be directed to the corresponding authors.
